# Supplementary material for: Pharmacogenomics of Vincristine-Induced Peripheral Neuropathy in Children with Cancer: A Systematic Review and Meta-Analysis
Source: Cancers (Basel). 2022 Jan 26;14(3):612. doi: 10.3390/cancers14030612 (PMC8833506; doi:10.3390/cancers14030612)

Supplementary materials

Supplementary materials 1 – Search strategies

Search strategy for PubMed (14 December 2020)

Search	Query	Results
#5	#1 AND #2 AND #3 AND #4	**175**
#4	"Complex Regional Pain Syndromes"[Mesh] OR "Chronic Pain"[Mesh] OR "Neuralgia"[Mesh:noexp] OR "Nociceptive Pain"[Mesh:noexp] OR "Pain, Intractable"[Mesh] OR "Constipation"[Mesh] OR "Urinary Bladder, Neurogenic"[Mesh] OR "Paresthesia"[Mesh] OR "Reflex, Stretch"[Mesh] OR "Extremities"[Mesh] OR "Perception"[Mesh:noexp] OR "Differential Threshold"[Mesh] OR "Pain Threshold"[Mesh] OR "Touch"[Mesh] OR "Touch Perception"[Mesh] OR "Neural Conduction"[Mesh] OR "Vibration"[Mesh] OR (("peripher*"[tiab] OR "extremit*"[tiab] OR "bladder*"[tiab]) AND ("pain"[tiab] OR "neuropath*"[tiab] OR "neurotox*"[tiab] OR "disabilit*"[tiab] OR "neurodyn*"[tiab] OR "nerve pain*"[tiab] OR ("nervous"[tiab] AND ("disorder*"[tiab] OR "disease*"[tiab])) OR "nociceptive*"[tiab] OR "somatic"[tiab] OR "neuralgi*"[tiab] OR "neurogen*"[tiab])) OR "constipat*"[tiab] OR "numbness*"[tiab] OR "tingling*"[tiab] OR "paresthesi*"[tiab] OR "dysesthesi*"[tiab] OR "formicat*"[tiab] OR "stretch reflex*"[tiab] OR "tendon reflex*"[tiab] OR "knee reflex*"[tiab] OR "achilles reflex*"[tiab] OR "complex regional pain syndrome*"[tiab] OR "cprs"[tiab] OR "intractable pain*"[tiab] OR "refractory pain*"[tiab] OR ("perceptive"[tiab] AND "threshold*"[tiab]) OR "sensibilit*"[tiab] OR "vibrati*"[tiab] OR "oscillati*"[tiab] OR ("sensory"[tiab] AND "nerve*"[tiab]) OR "tactil*"[tiab] OR "touch*"[tiab] OR "tickl*"[tiab] OR "toxicit*"[tiab] OR "toxicity" [Subheading] OR "adverse effects" [Subheading] OR (("adverse*"[tiab] OR "side"[tiab]) AND ("effect*"[tiab] OR "event*"[tiab]))	**3,663,304**
#3	"Vincristine"[Mesh] OR "vinca alkaloid*"[tiab] OR "vinblastin*"[tiab] OR "vincaleukoblastin*"[tiab] OR "vinblastinsulfat*"[tiab] OR "velban*"[tiab] OR "velbe"[tiab] OR "cellblastin*"[tiab] OR "lemblastin*"[tiab] OR "vincamin*"[tiab] OR "vincimax"[tiab] OR "pervincamin*"[tiab] OR "vincapront*"[tiab] OR "cerebroxin*"[tiab] OR "devincan*"[tiab] OR "vincristin*"[tiab] OR "leurocristin*"[tiab] OR "citomid*"[tiab] OR "oncovin*"[tiab] OR "onkocristin*"[tiab] OR "vintec"[tiab] OR "vincrisul"[tiab] OR "cellcristin*"[tiab] OR "vincasar"[tiab] OR "farmistin*"[tiab] OR "vindesin*"[tiab] OR "desacetylvinblastine amide"[tiab] OR "eldisin*"[tiab] OR "enison*"[tiab] OR "cytocristin*"[tiab] OR "krebin*"[tiab] OR "marqibo"[tiab] OR "neocristin*"[tiab] OR "nevexitin*"[tiab] OR "nsc 67574"[tiab] OR "nsc67574"[tiab] OR "onco tcs"[tiab] OR "vincrex"[tiab] OR "vincrina"[tiab] OR "vinracin*"[tiab] OR "vin cristin*"[tiab]	**42,451**
#2	"child*"[tw] OR "schoolchild*"[tw] OR "infan*"[tw] OR "adolescen*"[tw] OR "pediatri*"[tw] OR "paediatr*"[tw] OR "neonat*"[tw] OR "boy"[tw] OR "boys"[tw] OR "boyhood"[tw] OR "girl"[tw] OR "girls"[tw] OR "girlhood"[tw] OR "youth"[tw] OR "youths"[tw] OR "baby"[tw] OR "babies"[tw] OR "toddler*"[tw] OR "teen"[tw] OR "teens"[tw] OR "teenager*"[tw] OR "newborn*"[tw] OR "postneonat*"[tw] OR "postnat*"[tw] OR "puberty"[tw] OR "preschool*"[tw] OR "suckling*"[tw]	**4,466,735**

	OR "picu"[tw] OR "nicu"[tw] OR "Arthritis, Juvenile"[Mesh] OR "Myoclonic Epilepsy, Juvenile"[Mesh] OR "Leukemia, Myelomonocytic, Juvenile"[Mesh] OR "Xanthogranuloma, Juvenile"[Mesh] OR "Juvenile Delinquency"[Mesh] OR "Corneal Dystrophy, Juvenile Epithelial of Meesmann"[Mesh]	
#1	"Pharmacogenetics"[Mesh] OR "Pharmacokinetics"[Mesh] OR "Genome-Wide Association Study"[Mesh] OR "Whole Exome Sequencing"[Mesh] OR "Area Under Curve"[Mesh] OR "Polymorphism, Single Nucleotide"[Mesh] OR "Polymorphism, Genetic"[Mesh] OR "Genomic Structural Variation"[Mesh] OR "DNA Copy Number Variations"[Mesh] OR "CEP72 protein, human"[Supplementary Concept] OR "CYP3A5 protein, human" [Supplementary Concept] OR "RALBP1 protein, human" [Supplementary Concept] OR "ACTG1 protein, human"[Supplementary Concept] OR "ATP Binding Cassette Transporter, Subfamily B"[Mesh] OR "CYP3A5 protein, human"[Supplementary Concept] OR "Metabolic Clearance Rate"[Mesh] OR "whole genome association analysis"[tiab] OR "pharmacogenet*"[tiab] OR "pharmacogenom*"[tiab] OR "pharmacokinet*"[tiab] OR "drug kinet*"[tiab] OR "genome wide association*"[tiab] OR "gwa"[tiab] OR "gwas"[tiab] OR "exome sequenc*"[tiab] OR "whole transcriptome sequenc*"[tiab] OR "transcriptome sequenc*"[tiab] OR "area under curve*"[tiab] OR "area under the curve*"[tiab] OR "auc"[tiab] OR "aucs"[tiab] OR "single nucleotide polymorph*"[tiab] OR "snp"[tiab] OR "snps"[tiab] OR "genetic polymorphism*"[tiab] OR "genomic structural varia*"[tiab] OR "genome structural varia*"[tiab] OR "copy number vari*"[tiab] OR "copy number polymorph*"[tiab] OR "copy number chang*"[tiab] OR "cep72"[tiab] OR "cytochrome p 450*"[tiab] OR "erythromycin n demethylase"[tiab] OR "cyp3a*"[tiab] OR "cytochrome p450*"[tiab] OR "taurochenodeoxycholate 6 alpha monooxygenase"[tiab] OR "atp binding cassette transport*"[tiab] OR "multidrug resistance protein*"[tiab] OR "p glycoprotein*"[tiab] OR "abcc1"[tiab] OR "abcb1"[tiab] OR "abcc2"[tiab] OR "abcc3"[tiab] OR "abcc10"[tiab] OR "rablp1"[tiab] OR "actg1"[tiab] OR "map4"[tiab] OR "capg"[tiab] OR "tubb1"[tiab] OR "tubb2*"[tiab] OR "tubb3"[tiab] OR "tubb4"[tiab] OR "tissue distributi*"[tiab] OR "metabolic clearanc*"[tiab] OR "total body clearanc*"[tiab]	**959,409**

Search strategy for Embase.com (14 December 2020)

No.	Query	Results
#7	#5 NOT #6	**1188**
#6	#5 AND ('conference abstract'/it OR 'conference paper'/it)	**247**
#5	#1 AND #2 AND #3 AND #4	**1435**
#4	'complex regional pain syndrome'/exp OR 'neuralgia'/de OR 'chronic pain'/exp OR 'nociceptive pain'/exp OR 'intractable pain'/exp OR 'constipation'/exp OR 'neurogenic bladder'/exp OR 'paresthesia'/exp OR 'myotatic reflex'/exp OR 'limb'/exp OR 'perception'/exp OR 'differential threshold'/exp OR 'pain threshold'/exp OR 'touch'/exp OR 'nerve conduction'/exp OR 'vibration'/exp OR 'toxicity'/exp OR 'adverse event'/de OR 'adverse drug reaction'/exp OR 'constipat*':ti,ab,kw OR (('peripher*':ti,ab,kw OR 'extremit*':ti,ab,kw OR 'bladder*':ti,ab,kw) AND ('pain':ti,ab,kw OR 'neuropath*':ti,ab,kw OR 'neurotox*':ti,ab,kw	**3726212**

	OR 'disabilit*':ti,ab,kw OR 'neurodyn*':ti,ab,kw OR 'nerve pain*':ti,ab,kw OR ('nervous':ti,ab,kw AND ('disorder*':ti,ab,kw OR 'disease*':ti,ab,kw)) OR 'nociceptive*':ti,ab,kw OR 'somatic':ti,ab,kw OR 'neuralgi*':ti,ab,kw OR 'neurogen*':ti,ab,kw)) OR 'numbness*':ti,ab,kw OR 'tingling*':ti,ab,kw OR 'paresthesi*':ti,ab,kw OR 'dysesthesi*':ti,ab,kw OR 'formicat*':ti,ab,kw OR 'stretch reflex*':ti,ab,kw OR 'tendon reflex*':ti,ab,kw OR 'knee reflex*':ti,ab,kw OR 'achilles reflex*':ti,ab,kw OR 'complex regional pain syndrome*':ti,ab,kw OR 'cprs':ti,ab,kw OR 'intractable pain*':ti,ab,kw OR 'refractory pain*':ti,ab,kw OR (('perceptive' NEAR/3 'threshold*'):ti,ab,kw) OR 'sensibilit*':ti,ab,kw OR 'vibrati*':ti,ab,kw OR 'oscillati*':ti,ab,kw OR (('sensory' NEAR/3 'nerve*'):ti,ab,kw) OR 'tactil*':ti,ab,kw OR 'touch*':ti,ab,kw OR 'tickl*':ti,ab,kw OR ((('adverse*' OR 'side') NEAR/3 ('effect*' OR 'event*')):ti,ab,kw) OR 'toxicit*':ti,ab,kw	
#3	'vincristine'/exp OR 'vinblastine'/exp OR 'vinca alkaloid*':ti,ab,kw OR 'vinblastin*':ti,ab,kw OR 'vincaleukoblastin*':ti,ab,kw OR 'vinblastinsulfat*':ti,ab,kw OR 'velban*':ti,ab,kw OR 'velbe':ti,ab,kw OR 'cellblastin*':ti,ab,kw OR 'lemblastin*':ti,ab,kw OR 'vincamin*':ti,ab,kw OR 'vincimax':ti,ab,kw OR 'pervincamin*':ti,ab,kw OR 'vincapront*':ti,ab,kw OR 'cerebroxin*':ti,ab,kw OR 'devincan*':ti,ab,kw OR 'vincristin*':ti,ab,kw OR 'leurocristin*':ti,ab,kw OR 'citomid*':ti,ab,kw OR 'oncovin*':ti,ab,kw OR 'onkocristin*':ti,ab,kw OR 'vintec':ti,ab,kw OR 'vincrisul':ti,ab,kw OR 'cellcristin*':ti,ab,kw OR 'vincasar':ti,ab,kw OR 'farmistin*':ti,ab,kw OR 'vindesin*':ti,ab,kw OR 'desacetylvinblastine amide':ti,ab,kw OR 'eldisin*':ti,ab,kw OR 'enison*':ti,ab,kw OR 'cytocristin*':ti,ab,kw OR 'krebin*':ti,ab,kw OR 'marqibo':ti,ab,kw OR 'neocristin*':ti,ab,kw OR 'nevexitin*':ti,ab,kw OR 'nsc 67574':ti,ab,kw OR 'nsc67574':ti,ab,kw OR 'onco tcs':ti,ab,kw OR 'vincrex':ti,ab,kw OR 'vincrina':ti,ab,kw OR 'vinracin*':ti,ab,kw OR 'vin cristin*':ti,ab,kw	**134807**
#2	adolescen*:ti,ab,kw OR 'adolescence'/exp OR 'adolescent coping orientation for problem experiences'/exp OR 'adolescent development'/exp OR 'adolescent disease'/exp OR 'adolescent health'/exp OR 'adolescent parent'/exp OR 'adolescent pregnancy'/exp OR 'adolescent smoking'/exp OR 'adolescent'/exp OR 'adolescent-family inventory of life events and changes'/exp OR babies:ti,ab,kw OR baby:ti,ab,kw OR 'birth weight'/exp OR boy:ti,ab,kw OR boyhood:ti,ab,kw OR boys:ti,ab,kw OR 'brazelton neonatal behavioral assessment scale'/exp OR 'child abuse'/exp OR 'child advocacy'/exp OR 'child behavior checklist'/exp OR 'child behavior'/exp OR 'child care'/exp OR 'child death'/exp OR 'child health care'/exp OR 'child health'/exp OR 'child nutrition'/exp OR 'child parent relation'/exp OR 'child psychology'/exp OR 'child restraint system'/exp OR 'child safety'/exp OR 'child welfare'/exp OR child*:ti,ab,kw OR 'child'/exp OR 'childhood disease'/exp OR 'childhood mortality'/exp OR 'childhood'/exp OR girl:ti,ab,kw OR girlhood:ti,ab,kw OR girls:ti,ab,kw OR 'high risk infant'/exp OR infan*:ti,ab,kw OR 'infant disease'/exp OR 'infant mortality'/exp OR 'infant nutrition'/exp OR 'infant welfare'/exp OR 'infanticide'/exp OR 'infantile diarrhea'/exp OR 'infantile hypotonia'/exp OR 'juvenile delinquency'/exp OR neonat*:ti,ab,kw OR 'neonatal weight loss'/exp OR 'newborn disease'/exp OR 'newborn morbidity'/exp OR 'newborn period'/exp OR newborn*:ti,ab,kw OR 'newborn'/exp OR	**5857123**

	nicu:ti,ab,kw OR 'only child'/exp OR paediatr*:ti,ab,kw OR pediatr*:de,ab,ti,kw OR 'pediatric advanced life support'/exp OR 'pediatric anesthesia'/exp OR 'pediatric cardiology'/exp OR 'pediatric hospital'/exp OR 'pediatric intensive care nursing'/exp OR 'pediatric nurse practitioner'/exp OR 'pediatric nursing'/exp OR 'pediatric rehabilitation'/exp OR 'pediatric surgery'/exp OR 'newborn hypoxia'/exp OR 'pediatric ward'/exp OR 'pediatrics'/exp OR perinat*:ti,ab,kw OR 'perinatal development'/exp OR 'perinatal period'/exp OR 'persistent hyperinsulinemic hypoglycemia of infancy'/exp OR picu:ti,ab,kw OR postnat*:ti,ab,kw OR 'postnatal care'/exp OR 'postnatal development'/exp OR 'postnatal growth'/exp OR postneonat*:ti,ab,kw OR preschool*:ti,ab,kw OR puberty:ti,ab,kw OR 'runaway behavior'/exp OR 'school child':ti,ab,kw OR schoolchild*:ti,ab,kw OR 'severe myoclonic epilepsy in infancy'/exp OR suckling*:ti,ab,kw OR teen:ti,ab,kw OR teenager*:ti,ab,kw OR teens:ti,ab,kw OR toddler*:ti,ab,kw OR 'transient hypogammaglobulinemia of infancy'/exp OR youth:ti,ab,kw OR youths:ti,ab,kw	
**#1**	'pharmacogenomics'/exp OR 'pharmacokinetics'/exp OR 'genetic association study'/exp OR 'genetic procedures'/exp OR 'pharmacokinetic parameters'/exp OR 'genetic polymorphism'/exp OR 'genetic variation'/exp OR 'gene and nucleic acid parameters'/exp OR 'cep72 gene'/exp OR 'cyp3a5 gene'/exp OR 'cyp3a5 3 gene'/exp OR 'ralbp1 protein human'/exp OR 'actg1 gene'/exp OR 'actg1 protein'/exp OR 'abc transporter'/exp OR 'cyp3a5 protein human'/exp OR 'drug clearance'/exp OR 'whole genome association analys*':ti,ab,kw OR 'pharmacogenet*':ti,ab,kw OR 'pharmacogenom*':ti,ab,kw OR 'pharmacokinet*':ti,ab,kw OR 'drug kinet*':ti,ab,kw OR 'genome wide association*':ti,ab,kw OR 'gwa':ti,ab,kw OR 'gwas':ti,ab,kw OR 'exome sequenc*':ti,ab,kw OR 'whole transcriptome sequenc*':ti,ab,kw OR 'transcriptome sequenc*':ti,ab,kw OR 'area under curve*':ti,ab,kw OR 'area under the curve*':ti,ab,kw OR 'auc':ti,ab,kw OR 'aucs':ti,ab,kw OR 'single nucleotide polymorph*':ti,ab,kw OR 'snp':ti,ab,kw OR 'snps':ti,ab,kw OR 'genetic polymorphism*':ti,ab,kw OR ((genom* NEAR/3 'structural varia*'):ti,ab,kw) OR 'copy number vari*':ti,ab,kw OR 'copy number polymorph*':ti,ab,kw OR 'copy number chang*':ti,ab,kw OR 'cep72':ti,ab,kw OR 'cytochrome p 450*':ti,ab,kw OR 'erythromycin n demethylase':ti,ab,kw OR 'cyp3a*':ti,ab,kw OR 'cytochrome p450*':ti,ab,kw OR 'taurochenodeoxycholate 6 alpha monooxygenase':ti,ab,kw OR 'atp binding cassette transport*':ti,ab,kw OR 'multidrug resistance protein*':ti,ab,kw OR 'p glycoprotein*':ti,ab,kw OR 'abcc1':ti,ab,kw OR 'abcb1':ti,ab,kw OR 'abcc2':ti,ab,kw OR 'abcc3':ti,ab,kw OR 'abcc10':ti,ab,kw OR 'rablp1':ti,ab,kw OR 'actg1':ti,ab,kw OR 'map4':ti,ab,kw OR 'capg':ti,ab,kw OR 'tubb1':ti,ab,kw OR 'tubb2*':ti,ab,kw OR 'tubb3':ti,ab,kw OR 'tubb4':ti,ab,kw OR 'tissue distributi*':ti,ab,kw OR ((metabolic NEAR/3 clearanc*):ti,ab,kw) OR (('total body' NEAR/3 clearanc*):ti,ab,kw)	**4096847**

Search strategy for Clarivate Analytics/Web of Science Core Collection (14 December 2020)

Set	Query	Results
#5	#4 AND #3 AND #2 AND #1	**152**
#4	TOPIC: ((("peripher*" OR "extremit*" OR "bladder*") NEAR/3 ("pain" OR "neuropath*" OR "neurotox*" OR "disabilit*" OR "neurodyn*" OR "nerve pain*" OR ("nervous" NEAR/3 ("disorder*" OR "disease*")) OR "nociceptive*" OR "somatic" OR "neuralgi*" OR "neurogen*")) OR "constipat*" OR "numbness*" OR "tingling*" OR "paresthesi*" OR "dysesthesi*" OR "formicat*" OR "stretch reflex*" OR "tendon reflex*" OR "knee reflex*" OR "achilles reflex*" OR "complex regional pain syndrome*" OR "cprs" OR "intractable pain*" OR "refractory pain*" OR ("perceptive" NEAR/3 "threshold*") OR "sensibilit*" OR "vibrati*" OR "oscillati*" OR ("sensory" NEAR/3 "nerve*") OR "tactil*" OR "touch*" OR "tickl*" OR “toxicit*” OR (("adverse*" OR "side") NEAR/3 ("effect*" OR "event*")))	**1,989,313**
#3	TOPIC: ("vinca alkaloid*" OR "vinblastin*" OR "vincaleukoblastin*" OR "vinblastinsulfat*" OR "velban*" OR "velbe" OR "cellblastin*" OR "lemblastin*" OR "vincamin*" OR "vincimax" OR "pervincamin*" OR "vincapront*" OR "cerebroxin*" OR "devincan*" OR "vincristin*" OR "leurocristin*" OR "citomid*" OR "oncovin*" OR "onkocristin*" OR "vintec" OR "vincrisul" OR "cellcristin*" OR "vincasar" OR "farmistin*" OR "vindesin*" OR "desacetylvinblastine amide" OR "eldisin*" OR "enison*" OR "cytocristin*" OR "krebin*" OR "marqibo" OR "neocristin*" OR "nevexitin*" OR "nsc 67574" OR "nsc67574" OR "onco tcs" OR "vincrex" OR "vincrina" OR "vinracin*" OR "vin cristin*")	**29,876**
#2	TOPIC: ("adolescen*" OR "babies" OR "baby" OR "boy" OR "boyhood" OR "boys" OR "child*" OR "girl" OR "girlhood" OR "girls" OR "infan*" OR "neonat*" OR "newborn*" OR "nicu" OR "paediatr*" OR "pediatr*" OR "perinat*" OR "picu" OR "postnat*" OR "postneonat*" OR "preschool*" OR "puberty" OR "schoolchild*" OR "suckling*" OR "teen" OR "teenager*" OR "teens" OR "toddler*" OR "youth" OR "youths")	**3,091,920**
#1	TOPIC: ("whole genome association analys*" OR "pharmacogenet*" OR "pharmacogenom*" OR "pharmacokinet*" OR "drug kinet*" OR "genome wide association*" OR "gwa" OR "gwas" OR "exome sequenc*" OR "whole transcriptome sequenc*" OR "transcriptome sequenc*" OR "area under curve*" OR "area under the curve*" OR "auc" OR "aucs" OR "single nucleotide polymorph*" OR "snp" OR "snps" OR "genetic polymorphism*" OR ("genom*" NEAR/3 "structural varia*") OR "copy number vari*" OR "copy number polymorph*" OR "copy number chang*" OR "cep72" OR "cytochrome p 450*" OR "erythromycin n demethylase" OR "cyp3a*" OR "cytochrome p450*" OR "taurochenodeoxycholate 6 alpha monooxygenase" OR "atp binding cassette transport*" OR "multidrug resistance protein*" OR "p glycoprotein*" OR "abcc1" OR "abcb1" OR "abcc2" OR "abcc3" OR "abcc10" OR "rablp1" OR "actg1" OR "map4" OR "capg" OR "tubb1" OR "tubb2*" OR "tubb3" OR "tubb4" OR "tissue distributi*" OR ("metabolic" NEAR/3 "clearanc*") OR ("total body" NEAR/3 "clearanc*"))	**690,958**

Supplementary materials 2 – Data extraction template

The following study characteristics were extracted:

- Author and year of publication;
- Study design;
- Number of participants with VIPN and genotype data available;
- Treatment protocol;
- Method used for VIPN assessment.

The following baseline characteristics were extracted:

- Age;
- Sex;
- Race/ethnicity;
- Disease of patients;
- Dosage of VCR;
- Cumulative dosage of VCR;
- Prevalence of VIPN;
- Treatment phase in which VIPN was assessed (for patients with ALL).

The following data were extracted for the systematic review and meta-analysis:

- Minor allele frequency;
- Definition of cases and controls with VIPN per genotype-phenotype association;
- Method of calculating effect size;
- Effect size with 95% confidence intervals (if applicable);
- Effect: protective or risk.

The following data were extracted for the meta-analysis:

- Number of patients per genotype group with and without VIPN.

Supplementary tables

Table S1. Risk of bias of the included studies.

Authors and year	Selection bias	Study design	Confounders	Blinding	Data collection methods	Withdrawals and drop-outs	Analyses	Selection reported results	Global rating
Abaji et al. 2018 [52]	Moderate	Strong	Strong	Moderate	Moderate	Moderate	Strong	Strong	Strong
Abo-Bakr et al. 2017 [47]	Strong	Moderate	Moderate	Weak	Strong	Strong	Strong	Strong	Moderate
Aplenc et al. 2003 [28]	Strong	Moderate	Moderate	Weak	Strong	Weak	Strong	Strong	Moderate
Ceppi et al. 2014 [8]	Strong	Strong	Strong	Moderate	Moderate	Strong	Strong	Strong	Strong
Diouf et al. 2015 [9]	Weak	Strong	Moderate	Weak	Strong	Strong	Strong	Strong	Moderate
Egbelakin et al. 2011 [29]	Strong	Moderate	Moderate	Strong	Moderate	Strong	Strong	Strong	Strong
Guilhaumou et al. 2011 [20]	Moderate	Moderate	Moderate	Weak	Strong	Strong	Strong	Strong	Moderate
Gutierrez-Camino et al. 2016 [10]	Strong	Moderate	Strong	Strong	Moderate	Strong	Strong	Strong	Strong
Gutierrez-Camino et al. 2017 [48]	Strong	Moderate	Moderate	Strong	Moderate	Strong	Strong	Strong	Strong
Kayilioğlu et al. 2017 [30]	Moderate	Moderate	Moderate	Moderate	Moderate	Strong	Strong	Strong	Strong
Kishi et al. 2007 [13]	Strong	Moderate	Moderate	Weak	Moderate	Strong	Strong	Strong	Moderate
Li et al. 2019 [53]	Strong	Strong	Moderate	Weak	Strong	Strong	Strong	Strong	Moderate
Lopez-Lopez et al. 2016 [11]	Strong	Moderate	Strong	Strong	Moderate	Strong	Strong	Strong	Strong
Martin-Guerrero et al. 2019 [49]	Strong	Moderate	Strong	Strong	Moderate	Strong	Strong	Strong	Strong
McClain et al. 2018 [31]	Strong	Moderate	Moderate	Moderate	Moderate	Strong	Strong	Strong	Strong
Plasschaert et al. 2004 [22]	Moderate	Moderate	Moderate	Moderate	Moderate	Moderate	Strong	Strong	Strong

Renbarger et al. 2008 [14]	Strong	Moderate	Moderate	Moderate	Weak	Strong	Strong	Strong	Moderate
Sims et al. 2016 [32]	Moderate	Moderate	Moderate	Weak	Strong	Strong	Strong	Strong	Moderate
Skiles et al. 2018 [16]	Moderate	Moderate	Moderate	Weak	Strong	Strong	Strong	Strong	Moderate
Wright et al. 2019 [51]	Moderate	Moderate	Strong	Strong	Moderate	Strong	Strong	Strong	Strong
Zgheib et al. 2018 [50]	Strong	Moderate	Moderate	Moderate	Moderate	Strong	Strong	Strong	Strong

Table S2. Studies that assessed the effect of covariates on significant associations.

Author and year of publication	Assessed covariates	Association	Results multivariate analysis (OR + 95% CI)
Abaji et al. 2018 [52]	Age, treatment protocol, risk	SYNE2 (rs2781377)	2.7 (1.2-6.0)
		MRPL4 (rs10513762)	3.9 (1.5-10)
		BAHD1 (rs3803357)	0.3 (0.2-0.8)
Ceppi et al. 2014 [8]	Age, treatment protocol, risk group, BSA	ABCB1 (rs4728709)	0.3 (0.1-0.9)
		ACTG1 (rs1135989)	2.6 (1.1-6.0)
		CAPG (rs3770102)	0.07 (0.01-0.6)
Diouf et al. 2015 [9]	Genetically defined ancestry, cumulative vincristine dosage	MTNR1B (rs12786200)	Meta-analysis (combining St. Jude + COG), p = 6.30 × 10−7
		ETAA1 (rs17032980)	Meta-analysis (combining St. Jude + COG), p = 9.01 × 10−7
		TMEM215 (rs4463516)	Meta-analysis (combining St. Jude + COG), p = 3.02 × 10−8
		NDUFAF6 (rs7818688)	Meta-analysis (combining St. Jude + COG), p = 5.03 × 10−7
Kishi et al. 2007 [13]	Self-reported race, age, sex, treatment group	CYP3A5 expression	2.81 (1.24-6.39)
		VDR (rs1544410)	2.22 (1.06-4.67)
	Genetically defined ancestry, age, sex, treatment group	CYP3A5 expression	2.76 (1.34-5.71)
		VDR (rs1544410)	2.16 (1.03-4.51)
Lopez-Lopez et al. 2016 [11]	Age, sex, cumulative vincristine dosage	ABCB1 (rs10244266, rs10268314, rs10274587)<br>ABCC1 (rs1967120, rs3743527, rs11642957, rs11864374, rs12923345, rs17501331)<br>ABCC2 (rs12826, rs3740066, rs2073337, rs4148396, rs11190298	Covariates not significant (multivariate OR not reported)
Martin-Guerrero et al. 2019 [49]	Age, sex, cumulative vincristine dosage	MAPT (rs11867549)	Covariates not significant (multivariate OR not reported)
		miRNA-4481 (rs7896283)<br>miRNA-6076 (rs35650931)	
Wright et al. 2019 [51]	Sex, vincristine duration, nifedipine use, genetic ancestry	ABCC1 (rs3784867)	4.91 (1.99-12.10)
		SLC5A7 (rs1013940)	8.60 (1.68-44.15)

Table S3. Effect of CYP3A5 single-nucleotide polymorphisms (SNPs) on vincristine-induced peripheral neuropathy (VIPN). Reference allele is *1 (extensive metabolizer), variant alleles are *3 (rs776746), *6 (rs10264272), and *7 (rs41303343). Patients with at least one *1 allele are considered to be expressers of CYP3A5. Patients without *1 allele are considered to be non-expressers of CYP3A5. Recessive OR: non-expresser (*3/*3) compared to expresser (other genotypes).

Author and year of publication	MAF (%)	Definition cases VIPN	Definition controls	Method effect size	Univariate effect size with 95% CI (if applicable)	Effect
Included in meta-analysis						
Aplenc et al. 2003 [28]	*3: 11.8	Grade 3-4	Grade 0-2	Allelic OR	7.69 (1.20-333.33)	Risk[1]
Ceppi et al. 2014 [8]	Expresser: 13.5<br>Non-expresser: 86.5	Grade 3-4	Grade 0	Dominant OR	0.58 (0.24-1.38)	Not significant
Egbelakin et al. 2014 [29]	Expresser: 20.8<br>Non-expresser: 82.2	Grade 3-4	Grade 0	Dominant OR	2.26 (0.81-62.75)	Not significant
Guilhaumou et al. 2011 [20]	Expresser: 20.8<br>Non-expresser: 79.2	Global toxicity score ≥3	Global toxicity score <3	Dominant OR	1.44 (0.19-11.04)	Not significant
Kishi et al. 2007 [13]	Expresser: 32.1<br>Non-expresser: 67.9	Grade 2-4	Grade 0-1	Dominant OR	2.19 (1.13-4.26)	Risk
McClain et al. 2018 [31]	Expresser: 38.0<br>Non-expresser: 62.0	Grade 3-4	Grade 0-2	Dominant OR	0.75 (0.39-1.45)	Not significant
Renbarger et al. 2008 [14]	Caucasians (surrogate for non-expresser): 81.4<br>AAs (surrogate for expresser): 18.6	Grade 1-4	Grade 0	Dominant OR	10.67 (1.37-83.17)	Risk
Sims et al. 2016 [32]	Non-expresser: 16.7<br>Expresser: 83.3	Grade 1-3	Grade 0	Dominant OR	0.48 (0.05-4.56)	Not significant
Skiles et al. 2018 [16]	Expresser: 91.0<br>Non-expresser: 9.0	Grade 7 or higher	Grade 6 or lower	Dominant OR	1.95 (0.07-51.21)	Not significant
Not included in meta-analysis						
Kayilioğlu et al. 2018 [30]	Expresser: 15.2<br>Non-expresser: 84.8	Grade 2-4	Grade 0-1	Chi-square	P-value>0.05	Not significant

MAF = minor allele frequency, VIPN = vincristine-induced peripheral neuropathy, OR = odds ratio, CI = confidence interval, AAs = African-Americans.

Table S4. Raw data used to calculate odds ratios (OR) for the effect of CYP3A5 single-nucleotide polymorphisms (SNPs) on vincristine-induced peripheral neuropathy (VIPN). Reference allele is *1 (extensive metabolizer), variant alleles are *3 (rs776746), *6 (rs10264272), and *7 (rs41303343). Patients with at least one *1 allele are considered to be expressers of CYP3A5. Patients without *1 allele are considered to be non-expressers of CYP3A5. Dominant OR: non-expresser (*3/*3) compared to expresser (other genotypes).

Author and year of publication	CYP3A5 expression status	VIPN (n)	No VIPN (n)
Ceppi et al. 2014 [8]	Non-expresser	30	188
	Expresser	8	29
Egbelakin et al. 2014 [29]	Non-expresser	50	38
	Expresser	7	12
Guilhaumou et al. 2011* [20]	Non-expresser	2	3
	Expresser	6	13
McClain et al. 2018 [31]	Non-expresser	24	120
	Expresser	20	75
Renbarger et al. 2008 [14]	Caucasians (non-expressers)	32	60
	AAs (expressers)	1	20
Sims et al. 2016 [32]	Non-expresser	26	9
	Expresser	6	1
Skiles et al. 2018* [16]	Non-expresser	0	10
	Expresser	1	61

VIPN = vincristine-induced peripheral neuropathy, AAs = African-Americans.

* Additional data provided by the authors.

Figure S1. Funnel plot for evaluation of small study effects in the meta-analysis on the effect of CYP3A5 expression status on vincristine-induced peripheral neuropathy.

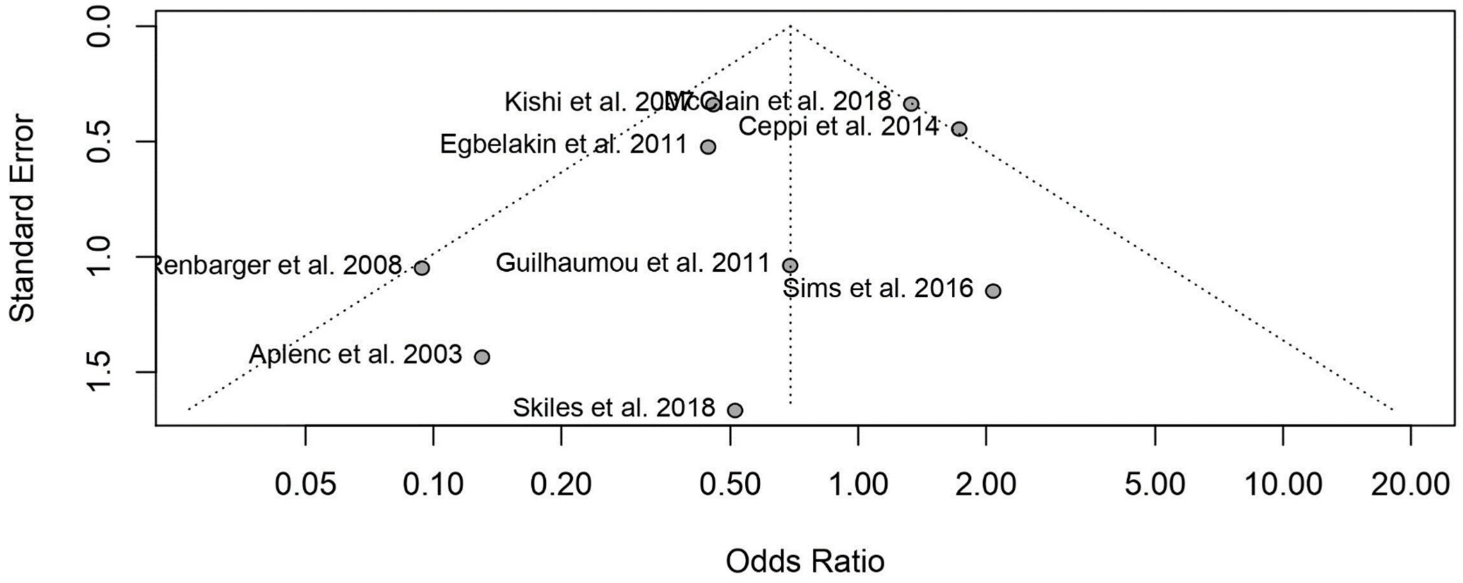

Supplement: Supplementary file 1 [file cancers-14-00612-s001.zip › Supplementary materials.pdf]
